# How Subtle Is the “Terroir” Effect? Chemistry-Related Signatures of Two “Climats de Bourgogne”

**DOI:** 10.1371/journal.pone.0097615

**Published:** 2014-05-23

**Authors:** Chloé Roullier-Gall, Marianna Lucio, Laurence Noret, Philippe Schmitt-Kopplin, Régis D. Gougeon

**Affiliations:** 1 Institut Universitaire de la vigne et du vin, Jules Guyot, UMR A 02.102 Procédés Alimentaires et Microbiologiques, Equipe Procédés Alimentaires et Physico Chimie, AgroSupDijon/Université de Bourgogne, Dijon, France; 2 Analytical BioGeoChemistry, Helmholtz Zentrum München, German Research Center for Environmental Health, Neuherberg, Germany; 3 Chair of Analytical Food Chemistry, Technische Universität München, Freising-Weihenstephan, Germany; University of Edinburgh, United Kingdom

## Abstract

The chemical composition of grape berries is influenced by various environmental conditions often considered to be representative of a “terroir”. If grapes from a given terroir are assumed to reflect this origin in their chemical compositions, the corresponding wine should also reflect it. The aim of this work was therefore to reveal the “terroir” expression within the chemodiversity of grapes and related wines, using ultrahigh-resolution mass spectrometry. Grapes and corresponding wines, from two distinct – though very proximate – terroirs of Burgundy were analyzed over three vintages (2010, 2011 and 2012). Ultrahigh-resolution mass spectrometry and ultra-high performance liquid chromatography were used as untargeted and targeted approaches to discriminate complex chemical fingerprints for vintages, classes (wines, skins or musts), and terroirs. Statistical analyses revealed that even if vintages have the most significant impact on fingerprints, the most significant terroir differences are seen in the grapes of a given vintage.

## Introduction

Wine, a beverage with a long tradition and high value, arises from a complex interplay between environmental, genetic and human factors. Metabolic compositions of grapes and related wines are complex, and they include primary (e.g., sugars, organic acids, amino acids) and secondary metabolites (e.g., flavonoids, anthocyannins, and other pigments). Although phenolic compounds play a major role[1], all of these compounds influence the quality and organoleptic character of wines[2]. Grape berries contain the major compounds contributing to flavour, resulting from metabolic changes that occur during the growth of grape berries up until harvest [3]. During winemaking and particularly during the alcoholic fermentation, these compounds will either disappear, be directly transferred to wine or react to form new molecules. Yeast-driven metabolism further contributes to the chemical enrichment of the wine through, for instance, the enzymatic liberation of volatile organic molecules responsible for the aroma of wine. Modern technologies have allowed for the identification of thousands of metabolites existing in exceedingly small quantities in wine, which are a consequence of microbiological processes, climatic conditions, viticultural and oenological practices [4].

The notion of terroir in viticulture precisely refers to this complex interplay of factors. It involves the vine and its environment, including phenology, geography, geology, pedology and the local climate of a vineyard, along with human activity[5]. If numerous authors have proposed varying definitions of the concept of terroir (Seguin[5], Vaudour[6], Riou [7] or van Leeuwen [8]), they all agree on its geographical dimension. On this basis, it could be proposed that if grape composition is marked by chemical fingerprints from a given terroir, wines made from these grapes should also reflect related fingerprints. Most analytical studies so far have tackled the question of “terroir” from the viticulture point of view, considering the impact of environmental factors on the quality of the grape or the wine[9], [10]. Several factors such as soil type, environmental, agricultural practices, climatic conditions, vine phenology or winemaking processes – all of them considered to contribute to the terroir effect –may indeed change the chemical composition of grapes and wine [11], [12], [17], [18]. Regarding the analytical tools, parameters such as isotopic ratios, trace element compositions, phenolic and/or volatile profiles and geological markers have been employed to determine the geographical origin of wines [8], [9], [10], [13]–[16].In any cases where wines exhibited a significant terroir effect, any differences were overshadowed by the vintage effect[18]. From a sampling point of view, all of the studies so far have considered grapes and/or wines from different local areas [17], [19]–[21], regions [17], [20], [22], [23] or even countries [14], [15], [24], [25]. However, grapes were in all cases either from distinct varieties or made by distinct winemakers [20], [26], which added an intrinsic variability among samples that was not necessarily related to genuine terroir effects. Indeed, Tarr [27] has shown that both the varietal character and the terroir influence the metabolome of grape berry. We have recently shown that a terroir effect on grapes and related wines could be demonstrated in the case of four distinct vineyards located 40 km apart and managed by the same producer [18]. It clearly appears that a robust methodology for the discrimination of terroirs using a single measurement system would be a great advance. Matching techniques now allow the analytical profile (all of the targeted analytical measurements together) of a wine to be used to predict its region of origin[14], [15], [24]. Non-targeted analytical tools, especially NMR spectroscopy[19], [26], [28]–[30] and FTICR mass spectrometry [2], [4], [31], [32] have been shown to be efficient methodologies. In this study, FTICR mass spectrometry is considered as a non-targeted metabolomics approach through the semi-quantitative description of all low molecular weight metabolites in a specified biological sample (wine) [4], [18], [27], [33]–[35].

The objective of this work was therefore to go beyond our previous work [18] and implement strategies to discriminate grapes and related wines from two distinct terroirs (vineyards) in two neighboring villages in the Côte de Nuits (Burgundy), separated by less than 2 km and managed by a unique vine grower/winemaker. We employ a single powerful untargeted analytical approach in addition to classic UHPLC targeted phenolic compounds. For each terroir, Pinot noir grapes from young and old vines along with corresponding wines were considered over three successive vintages (2010, 2011 and 2012). Here we show that through the direct analysis of grape extracts and related wines using Fourier Transform Ion Cyclotron Resonance Mass Spectrometry (FTICR-MS), it is possible to obtain the spectrum of thousands of metabolites that can ionise within a given mass range, and which provide the complex specific metabolic fingerprints of vineyards. It is a fast and reliable process especially applicable to high-throughput analysis and in combination with multivariate statistics it can be used to quantitatively distinguish between samples [36], [37].

## Materials and Methods

### Wine samples and preparation (Pinot noir wine, must and skin extracts)

This work was carried out on two distinct vineyards managed by the same producer. The first is in the village of Flagey-Echezeaux and will be referred to as GE and the second vineyard is in the village of Vosne Romanée and will be referred to as VR. Both the GE and the VR vineyards are characterized by clay limestone soils standing on 175 My calcareous basements. For each vineyard, three vintages (2010, 2011 and 2012) were considered.

Two distinct grape samples (GE and VR) were thus considered for a given vintage, and 100 Pinot noir berries were collected in duplicate (two distinct places for a given vineyard) at harvest for each of them. Musts and skins were separated by pressing berries using a laboratory-scale press, and skins were further dried on filter papers. Skin extracts were obtained by crushing (Ultra Turrax, IKA, Wilmington) twice in pure methanol (LC-MS grade). Mixtures were then centrifuged (10 min, 25400 *g*).

Must samples were obtained by solid phase extraction (C18-SPE cartridges; 100 mg.mL^−1^ Backer bond SPE columns) to remove ions and target the analysis of moderately polar to non-polar organic matter. The cartridges were conditioned with 1 mL methanol, followed by 1 mL acidified ultra-pure water (1.25% formic acid), and the must samples were passed through each cartridge by gravity. Musts were eluted with 500 µL of methanol and stored in vials at −20°C.

For the 2012 vintage, corresponding wines were collected immediately after the alcoholic fermentation, and three types of wine were collected: free run, press and blended wines.

### Targeted UHPLC analysis of phenolic compounds

An Acquity UPLC system (Waters, Milford, MA, USA) equipped with a model 2996 PDA detector was used for the analysis on BEHC18 column purchased from Waters (Eschborn, Germany). Under the optimised conditions, the column oven was thermostated at 30°C and the sample system at 8°C. The sample (10 µL) was injected via full-loop injection. We used water-methanol-formic acid 100∶5∶0.1 (v/v) as solvent A and methanol as solvent B with a flow rate to 0.25 mL.min^−1^. The optimized system consisted in a stepwise gradient as follows: from 3 to 5% B (0–4 min), 5 to 8% B (4–10 min), 8% B (10–12 min), 8 to 10% B (12–14), 10 to 15% B (14–17 min), 15 to 30.1% B (17–19 min), 30.1 to 38% B (19–21 min), 38 to 41% B (21–24 min), 41 to 50% B (24–30 min), 50 to 100% B (30–31 min), 100% B (31–31.5 min), 100 to 3% B (31.5–32.5 min), 3% B (32.5–35 min). Detection was performed at 280 nm, 305 nm, 320 nm and 360 nm, and the chromatographic characteristics were calculated with the Waters Empower software. Chemically pure standards of trans-resveratrol, gallic acid, hydroxytyrosol, gentisic acid, caffeic acid, coumaric acid, isoquercitrin, cis-piceid, quercitin, quercetin, catechin, (−)-epicatechin and malvidin were purchased from Sigma-Aldrich (St. Louis, MO, USA). The purity of all phenolic standards was greater than 95%. Individual stock solutions (1000 ppm) were prepared in pure methanol and kept at −20°C in the dark. A working solution was prepared daily by dilution with water. Calibration standards were freshly prepared on the day of analysis by diluting the appropriate working solution with initial middle phase solution. The range of concentration was selected in function of the sensitivity of the UHPLC-PDA for each polyphenol. As shown for trans-resveratrol (Figure S1A), highly satisfying correlation curves between standard concentrations (mg.L^−1^) and peak areas as detected by UPLC were recorded. Resveratrol concentrations shown in Figure S1 were measured in triplicate over two consecutive days for three different samples (NSG, CNV and SB), thus illustrating the very high reproducibility of the UPLC quantitation, and showing that changes in the UPLC response to individual analytes reflect differences in levels of these analytes. Error bars indicate standard deviations lower than 0.5%.

### FTICR-MS analysis

High-resolution mass spectra were acquired on a Bruker (BrukerDaltonics GmbH, Bremen, Germany) solariX Ion Cyclotron Resonance Fourier Transform Mass Spectrometer (FTICR-MS) equipped with a 12 Tesla superconducting magnet (Magnex Scientific Inc., Yarnton, GB) and a APOLO II ESI source (BrukerDaltonics GmbH, Bremen, Germany) in the negative ionisation mode. The negative ion mode fingerprint showed greater variety in the composition and abundance of components in the analysed wines and a smaller number of adducts, as well as higher resolution[38]. 20 µL of the samples were diluted in 1 ml of methanol prior to injection [39] and introduced into the microeletrospay source at a flow rate of 120 µL.h^−1^. Spectra were first externally calibrated by using clusters of arginine (10 mg.L^−1^ in methanol), and the accuracy attained. Further internal calibration was performed for each sample by using ubiquitous fatty acids, reaching accuracy values of less than 0.1 ppm in routine day-to-day measurement [40]. Spectra were acquired with a time domain of 4 mega words over a mass range of *m/z* 100 to 1000. 500 scans were accumulated for each sample. The FTICR mass spectra were exported to peak lists at a signal-to-noise ratio (S/N) of 2 and higher[41]. After calibration and peak alignment [37], the m/z values can be annotated with unambiguous elemental formulas by in-house written software, due to the ultrahigh resolution (R = 400.000 at *m/z* 400, differentiating two masses differing with the pass of an electron) and mass accuracy of 0.1 ppm (electron mass accuracy). In conjunction with an automated theoretical isotope pattern comparison, the generated formulas were validated by setting sensible chemical constraints (N rule; O/C ratio ≤1; H/C ratio ≤2n+2; element counts: C≤100, H≤200, O≤80, N≤3, S≤3) and mass accuracy window (set here at +/− 0.2 ppm). Up to several thousand such compositions could be calculated containing C, H, O, N and S elements and could then be represented using two-dimensional van Krevelen diagrams, which sort them onto two axes according, for instance, to H/C and O/C atomic ratios[41], [42].

### Statistical analyses

Data Normalization: row data (mass spectra) were normalized by log transformation (x-m)/σ in order to stabilize the variance between samples [4], [32], [43].

Hierarchical Clustering Analysis (HCA) was performed with the Hierarchical clustering explorer 3.5 sofltware (Maryland, USA) on the normalized data. Euclidian distances and average linkages were chosen to measure distance. This method allows samples to be grouped into homogeneous and distinct clusters, without imposing preliminary hypotheses on the data.

Principal Component Analysis (PCA) is another unsupervised method with the capacity to reduce the complexity of a multivariate dataset. Its goal is to extrapolate important information and display it as a set of new independent variables called principal components. This method, like Hierarchical clustering, discloses the similarity pattern of the observations or variables. A 95% cut-off of the frequencies was used to select the optimum number of principal components. Partial least square discriminant analysis (PLS-DA) models were used to extract the most discriminative metabolites, which were further manually checked within the spectra. m/z values with a variable importance in projection (VIP) value>2 and *p* values <0.05 (Wilcoxon-Mann-Whitney test) were considered as relevant. A model is considered acceptable for biological data if R^2^>0.7 and Q^2^>0.4 [43], [56], [57]. PCA and PLS-DA models were performed with the SIMCA 9.0 software (Umetrics, Sweden).

Two-dimensional van Krevelen diagrams were constructed using compositional networks (based on elemental compositions) and functional networks based on selected functional group equivalents enabling improved assignment option of elemental composition and classification of organic complexity with tuneable validation windows[42].

## Results and Discussion

A total of thirty-four different samples of Pinot noir from three vintages, and belonging to two areas, Flagey-Echezeaux (GE) and Vosne Romanée (VR), were analyzed. These samples were first analyzed for phenolic compounds using UHPLC. As already shown, phenolic concentration can differ from one local area to the other, and can therefore provide a basis for geographical discrimination [17], [44]. As an example, Table 1 shows the concentrations of phenolics (UHPLC) and corresponding intensities (FTICR-MS) for six distinct wines from the 2012 vintage.

**Table 1:** Comparison of phenolic concentrations between targeted and non-targeted analyses.

Sample	1	1	2	2	3	3	4	4	5	5	6	6
**Locality**	**VR**	**VR**	**VR**	**VR**	**VR**	**VR**	**GE**	**GE**	**GE**	**GE**	**GE**	**GE**
**Type of wine**	free run	free run	Press	Press	blending	blending	free run	free run	Press	Press	blending	blending
**Method**	**UPLC (mg/L)**	**FTICR/MS**	**UPLC (mg/L)**	**FTICR/MS**	**UPLC (mg/L)**	**FTICR/MS**	**UPLC (mg/L)**	**FTICR/MS**	**UPLC (mg/L)**	**FTICR/MS**	**UPLC (mg/L)**	**FTICR/MS**
**Gallic A.** (m/z 169.01425)	29.32	10057193	28.2	8706123	29.03	11085709	26.35	7941212	24.41	5891401	24.72	7184990
**Hydroxytyrosol** (m/z 153.05572)	17.44	nd.	17.35	nd.	17.04	nd.	13.01	nd.	12.22	nd.	12.4	nd.
**Gentisic A.** (m/z 153.01933)	0.91	nd.	0.87	nd.	0.71	nd.	0.76	nd.	0.64	nd.	0.72	nd.
**(+) Catechin** (m/z 289.07176)	127.42	311233664	121.96	277021952	122.28	283116672	118.6	195920048	109.68	142164352	110.36	223117312
**Caffeic A.** (m/z 179.03495)	2.61	38309168	2.61	33692936	2.61	34495036	2.13	35477540	1.96	23733444	2	34448404
**(−) Epicatechin** (m/z 289.07176)	71.02	311233664	70.53	277021952	69.94	283116672	72.24	195920048	68.57	142164352	69.43	223117312
**Coumaric A.** (m/z 163.04007)	0.28	5218280	0.25	4572683	0.28	4767820	0.28	5980091	0.23	3689097	0.22	4459198
**Malvidin** (m/z 330.0745)	321.31	nd.	287.77	nd.	294.7	nd.	317.72	nd.	265.75	nd.	280.97	nd.
**Isoquercitrin** (m/z 463.0882)	3.7	5254493	4.32	3922291	3.57	4709192	4.57	3022361	4.61	3043291	4.09	3586106
**Trans-resveratrol** (m/z 227.07137)	9.53	22897272	8.38	16212247	8.6	17808434	8.29	13690299	6.56	7062201	7.02	13120387
**Cis-Piceid** (m/z 389.12419)	2.41	11420214	2.46	12907573	2.32	11029681	2.79	10434416	2.64	6120783	2.53	10310404
**Quercitrin** (m/z 447.09329)	0.49	2517049	0.47	1892392	0.48	3177980	0.7	3475663	0.64	3124468	0.64	3029415
**Quercetin** (m/z 301.03536)	5.35	89854544	4.2	74599280	2.92	63515860	6.7	70674312	4.2	4168332	4.91	65365708

Phenolics concentration as analyzed with UPLC (mg.L^−1^) and intensity as analyzed with FTICR-MS, from two neighboring terroirs (VR  =  Vosne Romanée and GE  =  Flagey-Echezeaux) from the 2012 vintage.

**Figure 1:**
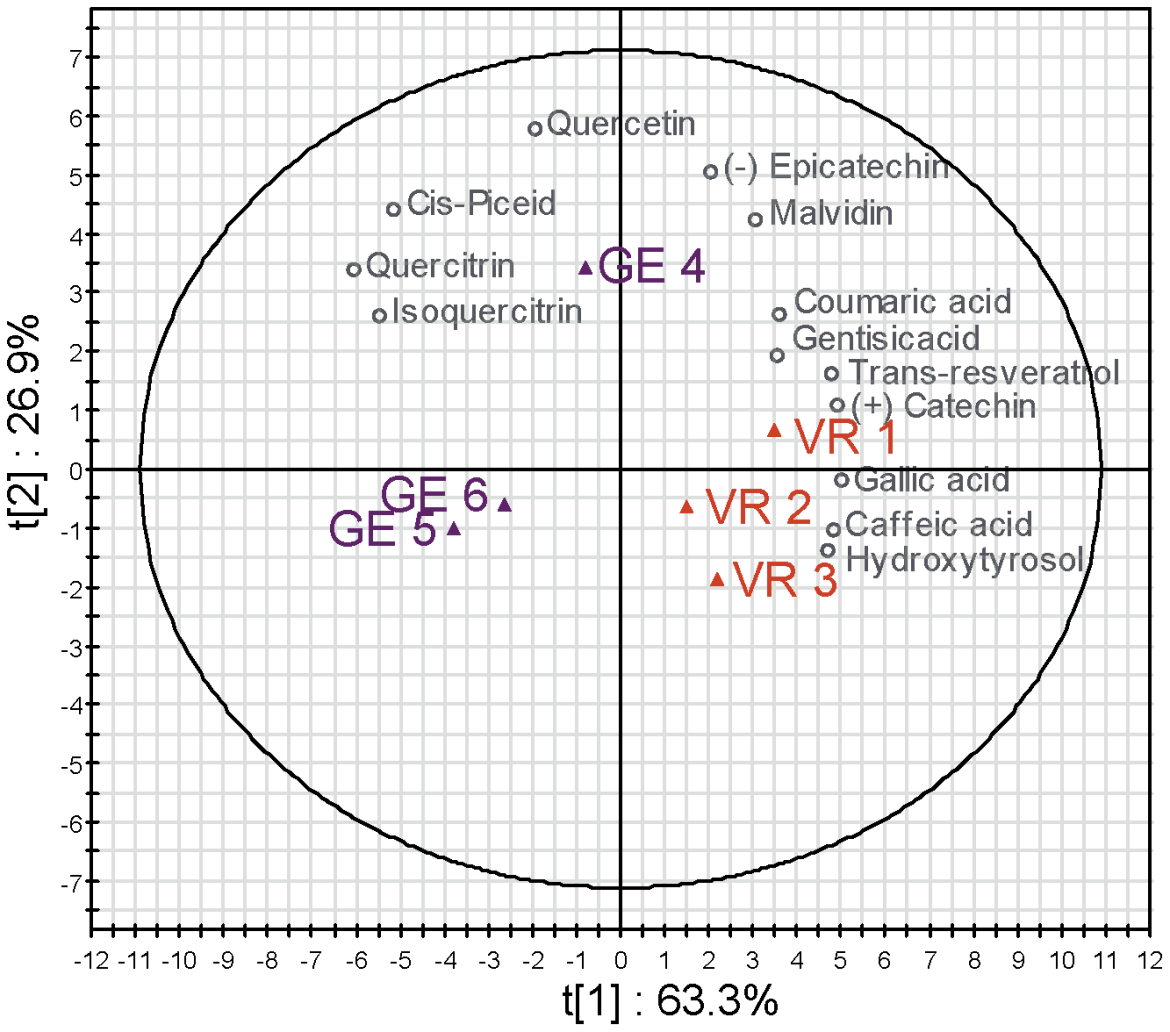
Statistical discrimination of wines according to phenolic concentrations. (A) Scores and loading plot for the PCA of UHPLC analyses. The first two components explain 90.2% of the variation. Color code: GE (purple), VR (orange), variables (grey).

The concentration of trans-resveratrol, for example, appeared to be high, averaging from 7.29 mg.L^−1^ (sample GE from 6.56 mg.L^−1^ to 8.29 mg.L^−1^) to 8.83 mg.L^−1^ (samples VR from 8.38 mg.L^−1^ to 9.53 mg.L^−1^). The different concentrations measured here agree with previously reported values for wines from several geographical origins, with levels of trans-resveratrol between 5 and 25 mg.L^−1^ [45], [46] and with results for three red wines from Burgundy (Figure S1B) which had levels of trans-resveratrol between 4 and 9 mg.L^−1^. Figure 1 shows the score and loading plots for the two first principal components (PC1 = 63.3%, PC2 = 26.9% of the total variance, respectively) for the phenolics concentrations of aforementioned six wines. Vosne Romanée wines (samples VR 1, 2 and 3) were clearly separated from Flagey-Echezeaux wines (samples GE 4, 5 and 6), and several compounds were found to be discriminant for this separation. Samples GE 4 and VR 1, which correspond to free run wine, seem to be distinguished from the other wines (press and mix) by higher concentrations of phenolic compounds, particularly in GE 4. By simultaneously considering the score and loading plots it is possible to interpret the variables that influence the positions of the observations in the score plots. VR wines were characterized by higher levels of gallic acid, hydroxytyrosol, gentisic acid, (+) catechin, caffeic acid, (−) epicatechin, coumaric acid, malvidin and resveratrol, whereas for GE wines, isoquercitrin, cis-piceid, quercitrin and quercetin were more abundant (Figure 1). These differences in concentrations are clear indications that, for the 2012 vintage, the accumulation of phenolic compounds in berries must have been influenced by environmental conditions, collectively referred to as terroir conditions, as illustrated for instance by trans-resveratrol[17], [29], [46]. Higher concentrations of trans-resveratrol in VR wines indeed suggest that vine stress within the last days before harvest was slightly more pronounced in the VR vineyard [47]. Similarly, higher concentrations of the fermentation-related hydroxytyrosol in VR wines indicate that the overall indigenous microbiology must have been different in the two vineyards[48].

**Figure 2:**
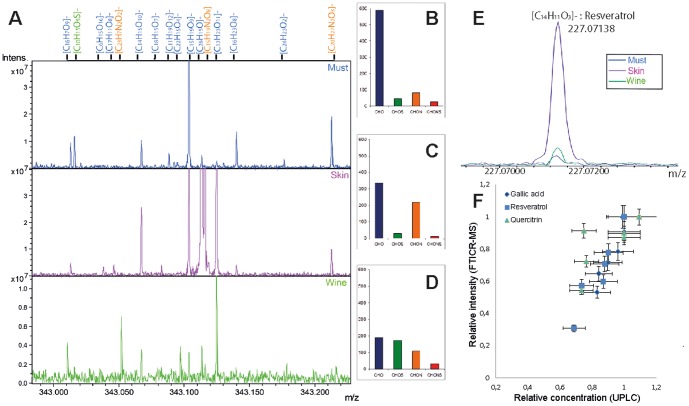
Detailed visualization of wine, must and skin extracts from the VR vineyard for the 2012 vintage, in the ESI(−) FTICR-MS. (A) spectra in the mass range m/z 343.000–343.200 with credible assignment of elemental formulas; Histograms of the relative frequency of (B) must, (C) skin and (D) wine (Color code: CHO, blue; CHOS, green; CHON, yellow and CHONS, red). (E) Zoom on the mass 227.07138 m/z, which corresponds to the [M–H]– ion with absolute mass formula [C_14_H_11_O_3_]– and can most likely be assigned to resveratrol isomers (F) Correlation between normalized concentrations from UHPLC and normalized peak intensities from FTICR-MS for resveratrol (square), gallic acid (diamond) and isoquercitrin (circle).

**Figure 3:**
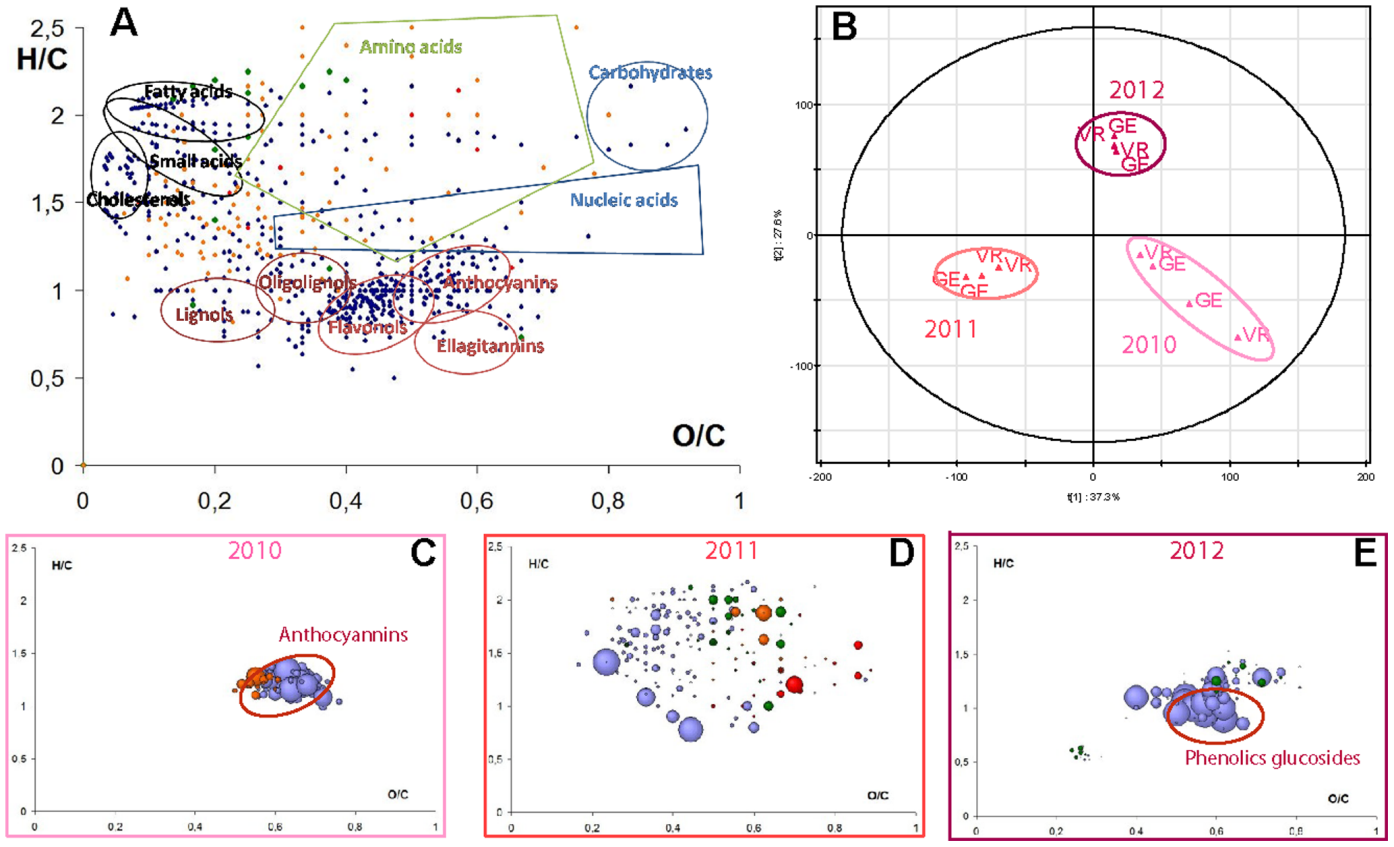
Differentiation of vintages and representations of related characteristic compounds. (A) H/C versus O/C van Krevelen diagram of all of the metabolites from our in-house database (MoG-DB), identifying regions specific to chemical families. (B) Scores plot for the PCA analysis of the negative-ion ESI FTICR-MS skin extracts data from both vineyards VR and GE for three different vintages; 2010 (pale), 2011 (medium) and 2012 (dark); The first two components explain 64.9% of the variation. H/C versus O/C van Krevelen diagrams of specific masses for (C) 2010, (D) 2011 and (E) 2012 vintages; (color code: CHO, blue; CHOS, green; CHON, red; CHONS, orange). Circle areas are proportional to mass peak abundance.

**Figure 4:**
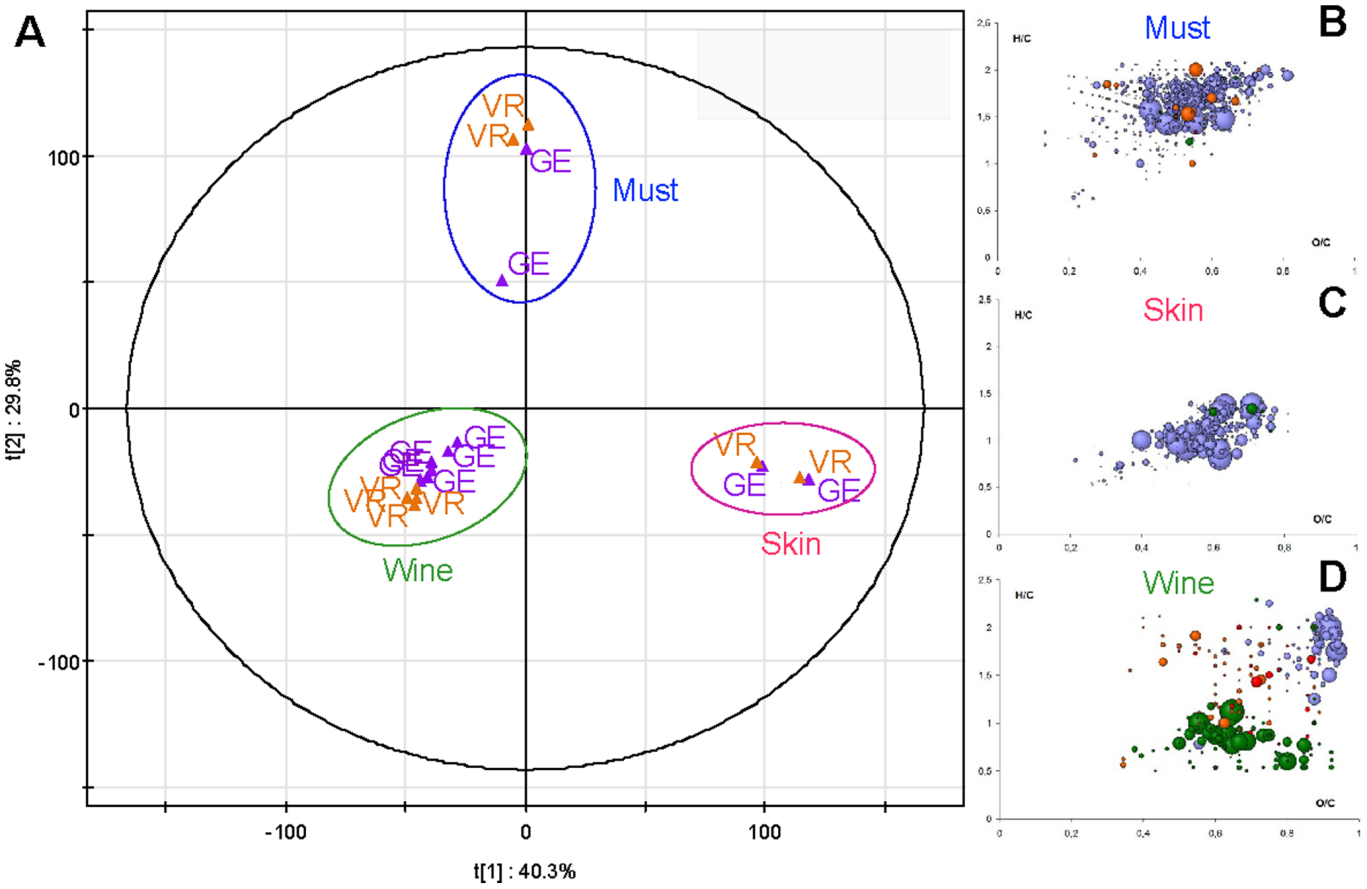
Differentiation of classes and representation of related characteristic compounds. (A) Scores plot for the PCA analysis of the negative-ion ESI FTICR-MS for wine, skin and must extract from the 2012 vintage and for the two villages VR (in orange) and GE (in purple). The first two components explain 70.1% of the variation. H/C versus O/C van Krevelen diagram of specific masses for (B) must, (C) skin and (D) wine. (Color code: CHO, blue; CHOS, green; CHON, red; CHONS, orange). Circle areas are proportional to mass peak abundance.

**Figure 5:**
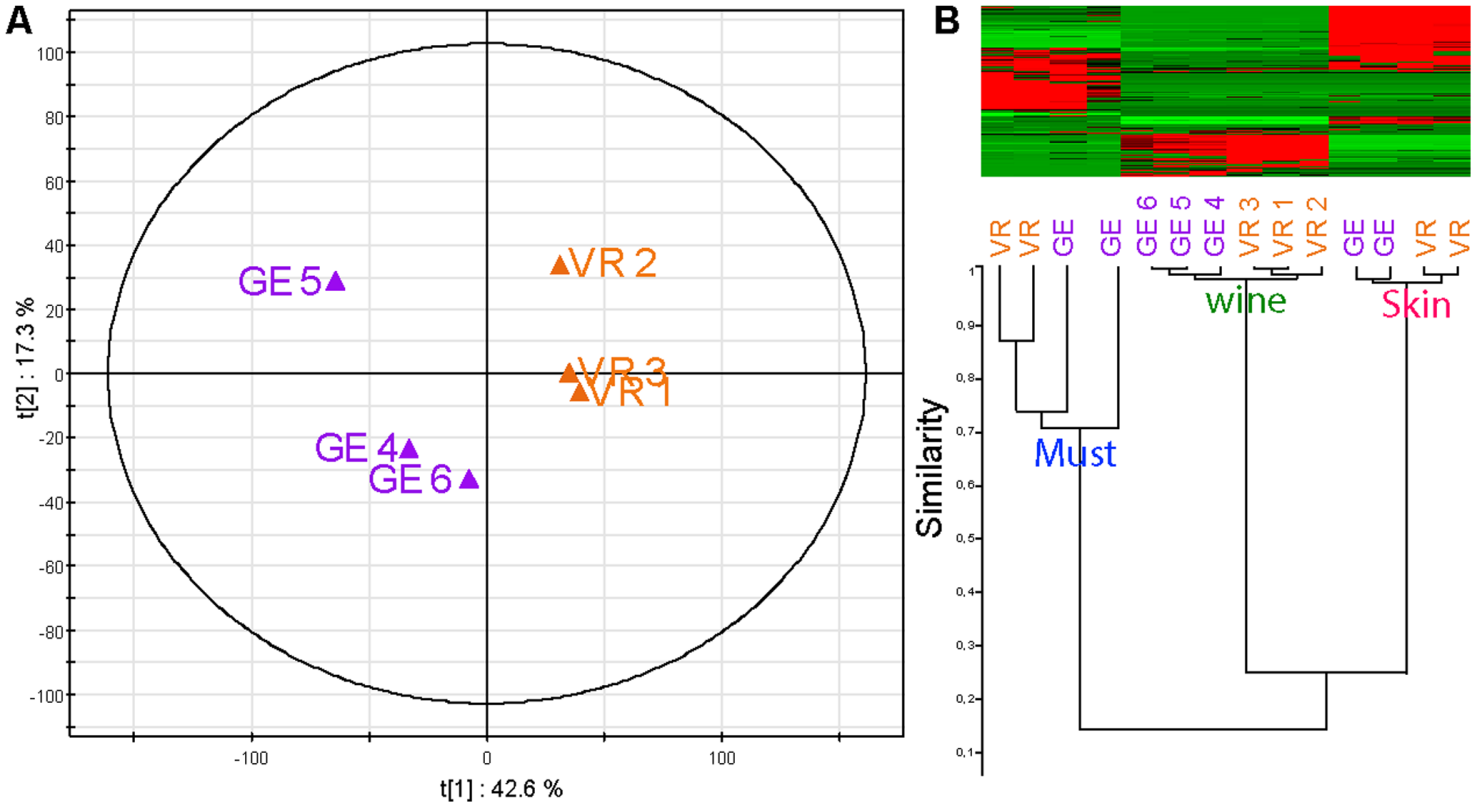
Terroir differentiation for the 2012 vintage. (A) Scores plot of the PCA analysis of the negative-ion ESIFTICR-MS wines data from VR (in orange) and GE (in purple) wines samples. The first two components explain 59.9% of the variation. (B) Hierarchical Cluster Analysis (HCA) of VR (in orange) and GE (in purple) Skin, wine and must samples are from 2012.

The phenolic profiles for both geographical areas were found to be quite distinctive within a single vintage. Indeed, the vintage effect on metabolic profiles of grapes and wines has been thoroughly studied[28], [49] and its importance is generally accepted. In order to have a more accurate view of the actual terroir-related biochemistry, which could potentially supersede any vintage effects, it is necessary to consider the largest possible number of metabolites. In Figures 2, 3, 4 and 5 we report the non-targeted metabolite analysis of a set of wines and grape extract samples. This approach, which uses multivariate statistics to analyze high-volume data sets, reveals the extremely high chemical diversity of grape and wine metabolites and offers the possibility to integrate the entire history metabolic changes throughout the elaboration process of wine[4]. We recorded the negative-ion ESI mode ICRFT-MS of each of 12 methanolic extracts from skins and musts (Figure 3A), and of 9 methanol-diluted wines. These samples were from three different vintages (2010, 2011 and 2012, only 2012 for wines) and from two nearby villages in the Côte de Nuits (GE and VR). FTICR-MS data were further statistically processed in order to identify discriminating m/z values. Metabolomics is considered here as the non-targeted metabolite analysis through semi-quantitative description of low molecular weight metabolites in wine and grape samples. The diversity of chemical spaces of wine and grape berries can be observed in the mass distributions within the 200 millimass range of a single nominal mass as illustrated for mass 343.00 (Figure 2A), where up to 16 possible elemental compositions based on C, H, O, N and S could be annotated (at S/N 4) for VR 2012 samples. 9 combinations were related to the must, 11 to grape skins and 8 to wine, with 2 being unique ([C_26_H_7_N_2_O_2_]– and [C_22_H_15_O_4_]–), demonstrating how the fermentation step can add to the biochemical diversity of a wine through the release of either nitrogen-containing or highly oxygenated compounds. The peak abundances and the distributions for CHO, CHOS, CHON and CHONS in these FTICR mass spectra were characteristic of wine- (Figure 2D), grape skin- (Figure 2C) or must- (Figure 2B) metabolites[32].The relative CHO abundance for the grape skins and musts was elevated, whereas it was lower for the wine. In contrast, the wine showed a relatively high abundance of S-containing compounds that could be due either to yeast secondary metabolites or to the addition of sulfites during winemaking. The peak at *m/z* 227.0713 (Figure 2E), which was significantly more intense in the must, corresponds to the [M–H]– ion with absolute mass formula [C_14_H_11_O_3_]– and could most likely be assigned to resveratrol isomers[4]. Such hypotheses could be supported by the comparison of relative peak intensities measured by FTICR-MS and UHPLC (Figure 2F). Indeed, relative intensities of FTICR-MS peaks corresponding to the elemental formulas of gallic acid, resveratrol and quercitrin were good matches to the various concentrations measured by UHPLC for these compounds (Figure 2F). Since such compounds are easily ionized under ESI conditions, Figure 2D confirms that the entire pool of compounds able to potentially be extracted from grapes is not necessarily found in the resulting wine.

The diversity of chemical spaces could be observed in the mass distribution and 2-dimentional van Krevelen diagrams (Figure 3A) were used to five an initial interpretation of such compilations of assigned elemental formulas. Using a home-compiled database of compounds that have been observed in wines, van Krevelen diagrams provide a representation of the specific contributions of the various phenolics, peptides, polysaccharides, nucleotides and any other classes of compounds present in wines that could be negatively ionized[4] (Figure 3A).We observe the specific distribution of elemental compositions (CHO, CHOS, CHON and CHONS) according to H/C and O/C atomic ratio. In grapes, chemical spaces from the four vineyards were more similar in composition to one another within a vintage than they were to chemical spaces of another vintage. Thus, as noted in our previous studies [18], only the discrimination based on vintages was clearly distinguished in the PCA scores plot (Figure 3B). This principal component analysis has demonstrated that individual samples corresponding to a given grape extract (skin in this case) could be clustered according to vintages regardless of the grapes' geographic origin, with the first axis accounting for most of the separation, although the 2012 vintage differentiation appeared to be also explained by the second axis (Figure 3B). This example illustrates that such a protocol can be used to analyse and generate reproducible results from individual grape and wine samples [50]. The projection of masses representing the characteristic chemical diversity associated with each of these three matrices (2010, 2011 and 2012) onto van Krevelen diagrams (Figure 3C-D-E) revealed that vintages were discriminated especially by CHO compounds. However, most interestingly, such CHO compounds appeared to be primarily located in the area containing the free polyphenolics for 2010, whereas they were primarily located in the zone containing glycosides in the 2012 samples. This illustrates how the vintage can impact the grape composition regardless of the geographical area. The discriminant chemical diversity for 2011 appeared to be surprisingly varied (Figure 3D). Proposed annotations for the discriminant masses (2010, 2011 and 2012 vintages for skin extracts) could be made using information from the literature and relevant databases. Particularly useful were an in-house database specific to plants and especially to grapes and wine, SciFinder Scholar, and the web server MassTRIX[51] (Table S1), which queries several databases (KEGG, Lipid Maps and HMDB). Annotations of peaks in the 2010 and 2012 samples indeed confirmed a relatively high occurrence of pertinent glycosidic structures in the 2012 vintage compared to 2010. It must be noted though that the hypothetical pelargonidin glucoside proposed for the 2012 vintage is an anthocyanin structure that has already been observed in *Vitis Vinifera* wines [52], but not from Pinot noir grapes. Metabolite annotations of the 2011 vintage spectra were characterized by pertinent hypothetical structures including sugars, phenolic acids and flavanols (Table S1).

If we consider wines and the distinct contributions from the grape skins and musts, from which they originated for a single vintage (here 2012), a PCA analysis of corresponding annotated mass peaks from both Flagey-Echezeaux and Vosne-Romanée vineyards clearly showed a distinct separation by the first two principal components, which explained 70.1% of the total variance (Figure 4A). For both the must and skin extracts, the composition appears to be more similar between the two vineyards than it is to the other extract (must or skin) from the same vineyard (musts in blue and skins in pink in Figure 4A). In agreement with Catharino [53], PCA also clearly separates musts (blue) from wines (green) into two well-defined groups, confirming the suitability of ESI-MS for the characterization of grape and wine chemical spaces. The discrimination between skin extracts and wines was mainly explained by the first component whereas the second component revealed the differentiation between skin extracts, on one hand, and wines and musts, on the other. The projection of specific data (masses as filtered from the PCA in Figure 4A) for the three classes on van Krevelen diagrams (Figure 4B–D), revealed that all three of the compartments: skin extracts, musts and wines, displayed highly rich and specific distributions of discriminant CHONS-containing elemental compositions (CHO, CHOS, CHON and CHONS). Must fingerprints were consistently related to CHO and CHON compounds corresponding to peptides, whereas skin samples were discriminated in particular on the basis of polyphenolic CHO compounds (Figure 4B–C). In contrast, compounds specific for wines appeared to be more diverse in terms of chemical families, including glycosylated CHO compounds, S-containing polyphenolic compounds and various CHON and CHONS compounds (Figure 4C). Two aspects of the van Krevelen signature for wines (Figure 4C) appear to be particularly interesting: the presence of sulfur-containing compounds (CHOS) especially in the aromatic area, and of the CHO compounds appearing in the top right corner of the diagram, indicating that carbohydrate-type compounds were specific for wines, whereas it has been shown that such compounds do not easily ionize under ESI conditions[54]. The former could be easily explained by the formation of S-adducts of polyphenols upon addition of sulfites during the winemaking process or by fermentation secondary metabolites, but an explanation for the latter is less straightforward. Finally, Figure 4A also revealed that each of the two villages (VR in orange and GE in purple) could be partly separated within the wine compartment, thus emphasizing latent terroir contributions in the chemodiversity of grapes and corresponding wines. With such high-resolution mass data, reliable structural assumptions could be drawn by querying topical databases on different annotated *Vitis vinifera* organism pathways, such as KEGG, accessible with the MassTRIX interface[51], [55] (Figure S2). 48 out of the 68 possible wine metabolites identified arise from the flavonoid biosynthesis pathway (see Figure S2A). Many of these metabolites are known to exist in wines and are therefore a reliable validation of such database querying using our raw sets of masses[4]. Applying a similar MassTRIX treatment to must, skin and wine led to the identification of 58, 51 or 48 distinct metabolites from the flavonoid biosynthesis-, fructose and mannose metabolism-, or fatty acid biosynthesis pathways, respectively, as shown in Figure S2B. As a whole, MassTRIX treatment of compounds that discriminate musts, skins and wines not only illustrates the possibility to consistently propose structural identifications for some of the compounds, but also shows that hits can be found for less than 10% of the discriminant masses when compared to the different *Vitis vinifera* pathways. Moreover, structures from existing related databases could be assigned to less than 20% of all of the detected signals, attesting to the magnitude of the structurally unresolved chemistry of wine[4].

**Figure 6:**
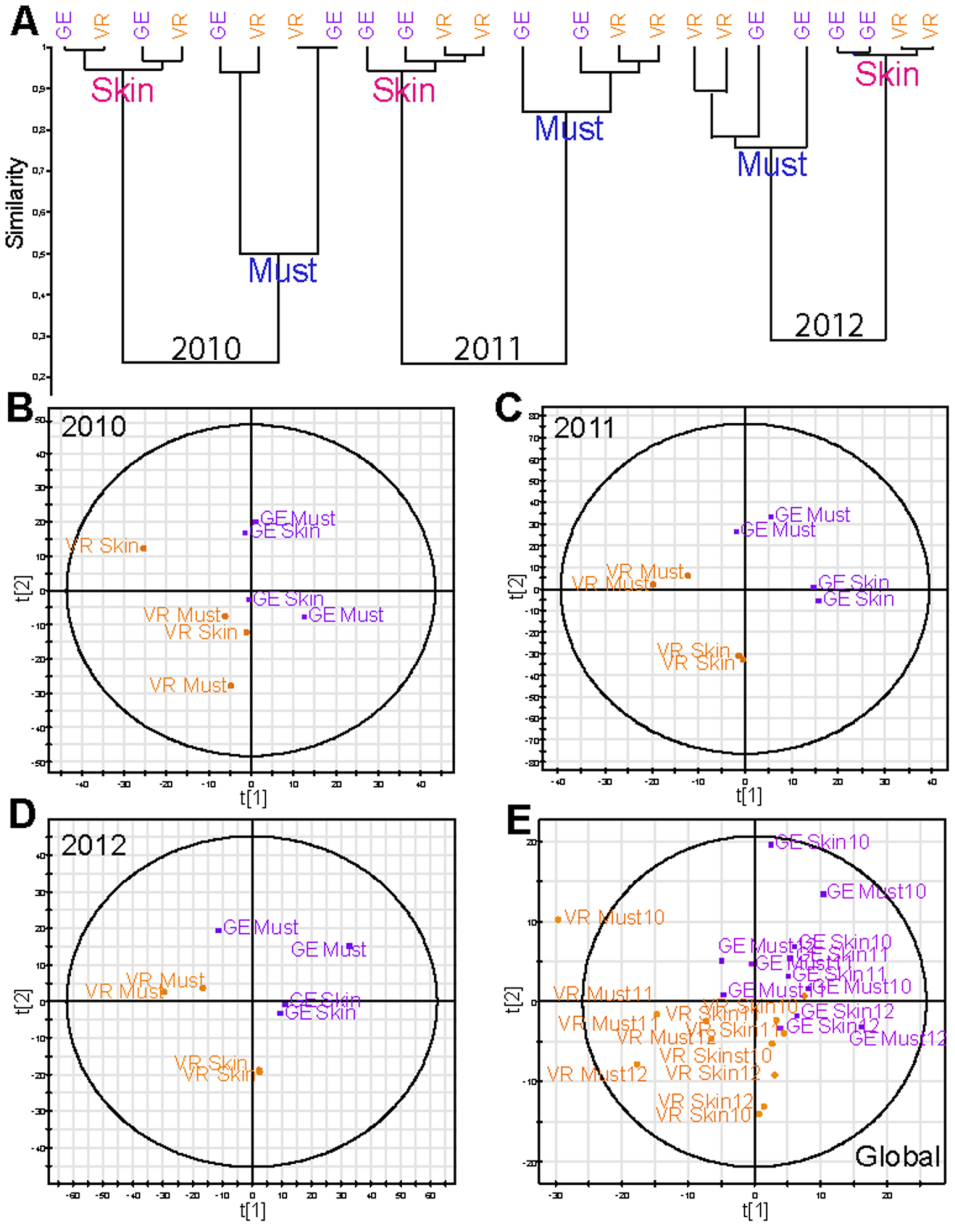
Terroir differentiation for skin and must samples from three different vintages (2010-2011-2012). (A) Hierarchical Cluster Analysis (HCA) of VR (in orange) and GE (in purple). Skin and must samples are from 2010, 2011 and 2012. Scores plot of the PLS-DA analysis of the negative-ion ESI FTICR-MS must and skin data from both vineyards VR and GE wines samples from (B) 2010 (C) 2011 (D) 2012 and (E) 2010–2011 and 2012.

When samples from both villages are considered for a given vintage (2012), terroir discriminations are immediately observed by PCA analysis of corresponding wine chemical spaces (Figure 5A). The first principal component accounts for the discrimination between wines from the two villages. It must be noted though, that the chemical space variability within GE wines appeared to be significantly higher than that observed for VR wines. Indeed, GE covers a larger vineyard area (2.29 ha) than VR (1.81 ha), which could account for the greater diversity amongst the GE samples. Hypothetical annotations of discriminant masses (VR and GE) could be obtained from the literature and relevant databases (Table S2), revealing characteristic structures as diverse as sugars, phenolic acids or fatty acids (Table S2). Most interestingly, the hierarchical cluster analysis of all the 2012 samples, including musts and skin extracts (Figure 5B) showed the excellent separation of the classes previously shown in Figure 4, and terroir discriminations were visible not only in the wine, but also in the grapes, with a stronger effect seen in musts than in skins and wines. Such results may suggest that winemaking processes could lead to some loss of terroir contributions, at least in young wines. It should be noted, that up to 7850 masses were recorded altogether for the different 2012 samples considered in Figure 5B of which 504 were discriminant for VR differentiation and 207 for GE. However, due to the limitations of current databases, very few relevant masses could be annotated. Based on these results, which show that some terroir impacts could potentially be stronger in grapes than in wines, HCA has been performed for each of the three vintages (Figure 6A) and PLS-DA analyses (Figure 6B-C-D-E) of musts and skin extracts for the two villages are presented. For each vintage, clusters not only clearly separate musts and skins (Figure 6A), but also consistently discriminate between the two villages for the 2011 and 2012 vintages, with a higher efficiency for musts as previously shown in Figure 5B-C-D. Nevertheless, such results clearly attest to significantly different environmental conditions (soil nature and biochemistry, climate, etc.) in VR and GE vines, which can be modulated by vintage effects, as exemplified by the 2010 vintage. This was further confirmed by PLS-DA on the same data sets, which distinctly discriminates between samples of the two villages, regardless of the vintage (Figure 6E), thus providing for the first time a clear representation of how “terroirs”, which can be as small as the numerous “climats de Bourgogne”, can actually give rise to grapes with significantly different chemical fingerprints. The two valid components of the model were obtained though seven-fold cross-validation with the following values: R^2^X(cum) = 0.91 and Q^2^(cum) = 0.46 for 2010, R^2^X(cum) = 0.98 and Q^2^(cum) = 0.77 for 2011 and R^2^X(cum) = 0.93 and Q^2^(cum) = 0.56 for 2012. These indices reaffirm the goodness of the fit and the prediction capacity of the model. This original result was uniquely confirmed by the PLS-DA taking into account the entire data set (musts and skin extracts) for the three vintages, which also separated the samples into two distinct groups of chemical fingerprints related to VR (in orange) and GE (in purple) terroirs regardless of the type of extract (musts and skin) or the vintage (Figure 6E).

## Conclusions

This study marks the first implementation of non-targeted analyses of grape extracts and corresponding wines from two neighboring villages in the Côte de Nuits, managed by a same producer, in order to assess discriminations based on terroir and vintage. Our results show that FTICR-MS spectra of grape extracts and wines can be used to compare terroirs as small as the numerous “Climats de Bourgogne” through their wine and grape chemodiversities. Our results therefore demonstrate that Pinot noir grapes grown in two distinct “Grands Crus” appellations separated by less than 2 km, have distinct chemical signatures of environmental conditions related to local climatic, geology, pedology and phenology characteristics, all contributing to the identification of the so-called “terroir”. This effect of terroir on metabolites is noticeable in wines, skin berries and especially musts. FTICR-MS allows the highest molecular resolution to date and thus the finest available visualization of the chemical composition that may be responsible for such fine discriminations. The first motivation of this manuscript was to highlight the chemical diversity in the wines, musts, and grape skins. Although we propose chemical structures based on the exact mass analysis, these identifications are only putative; further investigation is ongoing using UPLC-MS and tandem mass spectrometry complementary to the approach shown here to give conclusive structural identification of the metabolites of interest. Although the terroir effect was small compared to the variability induced by berry compartments or vintages, it could be significantly identified within individual vintages. Therefore, our results contribute to the representation of how wines – considered as pieces of art in terms of chemical equilibrium –bring messages from their birthplaces to the glass.

## Supporting Information

Figure S1 **Details of the UPLC analysis of resveratrol standards and wines: (A) Correlation curve between the concentration of resveratrol standards (mg.L^−1^) and the peak areas as detected by UPLC along with its calculated correlation coefficient (B) Histogram of resveratrol concentrations (mg.L^−1^) from three red wines from Burgundy (NSG, CNV and SB) resulting from three technical replicates, with standard deviation less than 0.5%.**
(TIF)

Figure S2 **Metabolic pathways of the** ***Vitis vinifera*** **organism as annotated from ICR-FT/MS data with the Masstrix translator into pathways for (A) Flavonoid biosynthesis pathway with annotated metabolites present in VR wines (B) Histogram plots of the number of annotations for various pathways (N) of VR skins (in pink), musts (in blue) and wines (in green).**
(TIF)

Table S1 **Examples of unique skin-specific masses from 2010, 2011 and 2012, number of known structures for each formula found by SciFinder scholar and putative annotations of known grape and wine metabolites.**
(XLSX)

Table S2 **Examples of unique wine-specific masses specific from VR and GE, number of known structures for each formula found by SciFinder scholar and putative annotations of known grape and wine metabolites.**
(XLSX)
