# Supplementary figures and images for: How Subtle Is the “Terroir” Effect? Chemistry-Related Signatures of Two “Climats de Bourgogne”
Source: PLoS One. 2014 May 23;9(5):e97615. doi: 10.1371/journal.pone.0097615 (PMC4032233; doi:10.1371/journal.pone.0097615)

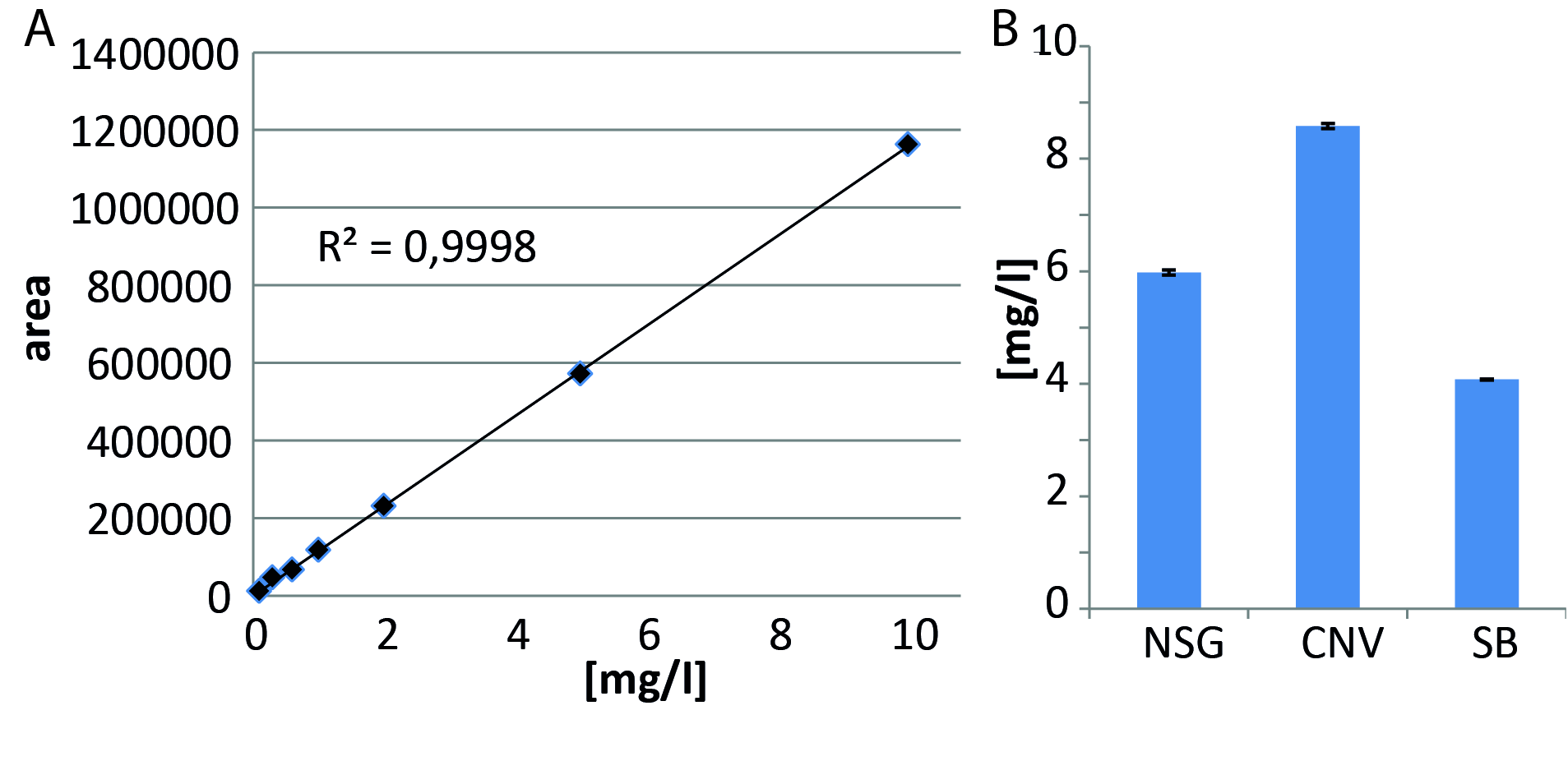

Supplement: Figure S1 — Details of the UPLC analysis of resveratrol standards and wines: (A) Correlation curve between the concentration of resveratrol standards (mg.L−1) and the peak areas as detected by UPLC along with its calculated correlation coefficient (B) Histogram of resveratrol concentrations (mg.L−1) from three red wines from Burgundy (NSG, CNV and SB) resulting from three technical replicates, with standard deviation less than 0.5%. (TIF) [file pone.0097615.s001.tif]

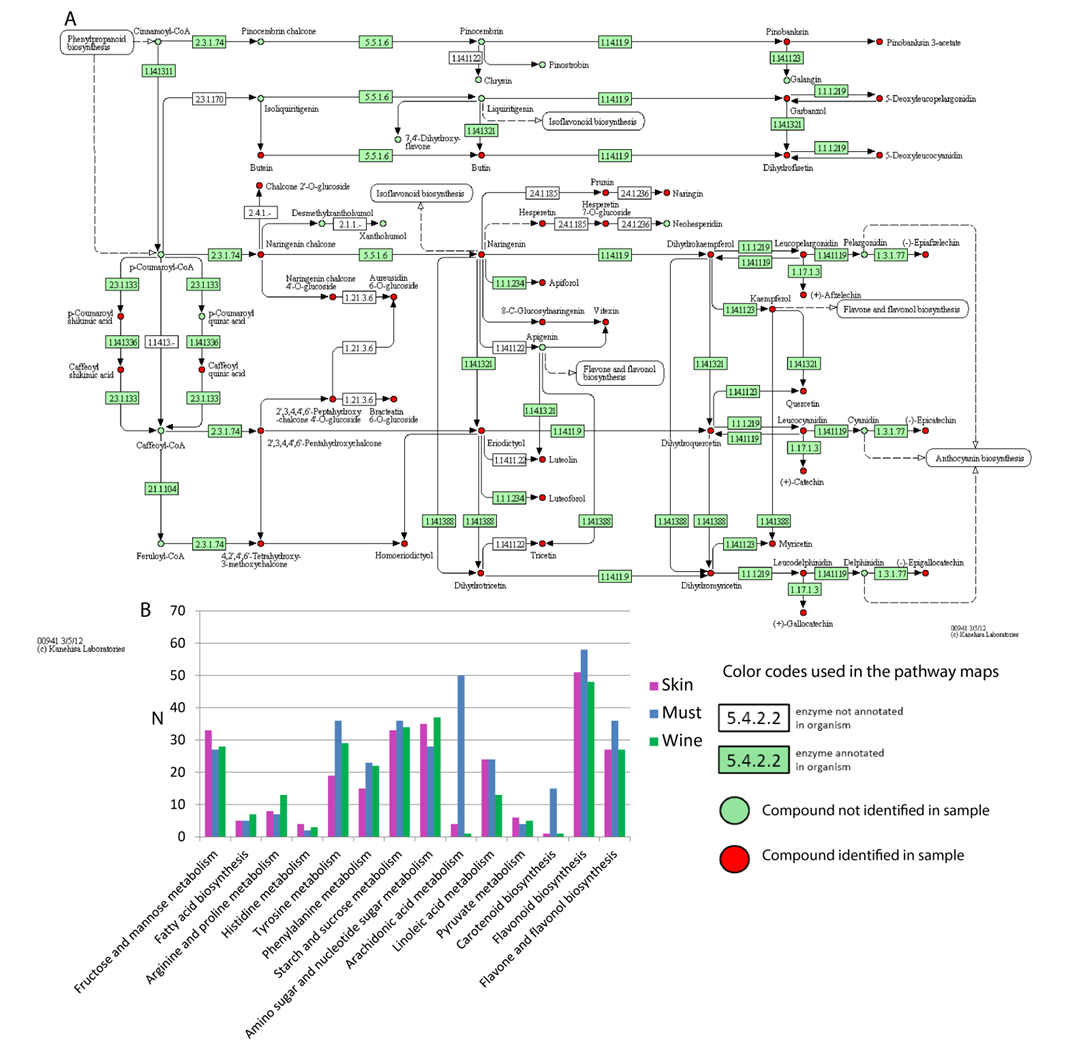

Supplement: Figure S2 — Metabolic pathways of the Vitis vinifera organism as annotated from ICR-FT/MS data with the Masstrix translator into pathways for (A) Flavonoid biosynthesis pathway with annotated metabolites present in VR wines (B) Histogram plots of the number of annotations for various pathways (N) of VR skins (in pink), musts (in blue) and wines (in green). (TIF) [file pone.0097615.s002.tif]
